# 

**Published:** 1914-03-14

**Authors:** 


### CONTENTS.

| | PAGE |
|---|---|
| **Specialism and Hospital Committees** ... ... | 633 |
| **Anti-Phthisis Activities in Berkshire** ... ... | 634 |
| **Hospital and Institutional News** ... ... ... | 635 |
| The Hospital's Special Number on Radium—Radium for the Withington Workhouse Hospital—A Legacy for the Asylums Board—The St. Albans and Mid-Herts Hospital—Domestic Servants as In-Patients — Smaller Hospitals and the Modern Standard—A Hospital Officer's Jubilee—A New Factor in Hospital Designs—Newcastle Workmen and the Truck Bill—Migration of the West End Hospital—Joint Sanatorium for Berks and Bucks—Illness of the Medical Superintendent of Croydon Infirmary—The Reconstruction of the Brownlow Hill Workhouse—A Blot on Poor-Law Administration—The New Middlesbrough Sanatorium—The Night Staff at Poor-Law Infirmaries—Public Opinion and Poor-Law Standards—A Cottage Hospital and Public Authorities—For Hospital Workers—The River Ambulance Service—Ireland and Venereal Disease—Finance and Developments at Brighton—The Salary of Infirmary Superintendents — Guardians and Medical Certificates—Effect of Mr. Samuel's Pronouncement—This Week's Drug Market—To Our Readers. | |
| **Modern Methods** ... ... ... ... ... | 641 |
| The Clinical Application of Hormones. | |
| **The Psycho-Therapeutics of Insanity** ... ... | 642 |
| By BERNARD HOLLANDER, M.D. | |
| **German Hospital Development** ... ... ... | 643 |
| An Expert Continental Review. | |
| **Research and Remedies** ... ... ... ... | 644 |
| **Reports on Hospitals of the United Kingdom**— | 645 |
| By SIR HENRY BURDETT, K.C.B., K.C.V.O | |
| Cranleigh Village Cottage Hospital. | |

[See over.]

### CONTENTS—(*continued*).

| | PAGE |
|---|---|
| **Personalia** ... ... ... ... ... ... ... | 646 |
| **National Insurance Act Developments** ... ... | 647 |
| Miscellaneous Notes and Comments. | |
| **The Origin and Evolution of the 18th Century Hospital Movement** ... ... ... ... | 649 |
| **Legal Notes for Institutional Workers** ... | 650 |
| Disease as an Accident. | |
| **Round the World** ... ... ... ... ... ... | 651 |
| Fellow-Workers and their Doings. | |
| **A London Ambulance Service** ... ... ... | 652 |
| The County Council's New Scheme Explained. | |
| **Non-Separated Infirmaries** ... ... ... ... | 653 |
| Dr. L. M. Guilding on the Effect of the Poor-Law Orders. | |
| **The Editor's Letter-Box** ... ... ... ... | 655 |
| Sanatoria and Convalescent Homes—Northampton General Hospital. | |
| **Appointments** ... ... ... ... ... ... ... | 655 |
| **Doctors' Wills** ... ... ... ... ... ... ... | 655 |
| **Buildings Contemplated** ... ... ... ... | 656 |
| **Gifts and Bequests** ... ... ... ... ... ... | 656 |
| **Institutional Needs** ... ... ... ... ... ... | 657 |
| **The Book Market** ... ... ... ... ... ... | 657 |
| **The National Medical Union (Official)** ... ... | 658 |
| Policies Favoured by Local Branches. | |
| **The Institutional Worker** ... ... (Suppl.) | 1 |
| I.—Duties of the Male Servants. | |
| Questions Invited. | |
| The Housekeepers' Department. | |
| Questions for March. | |
| Employment and Training. | |
| The Sick and in Need. | |
| Practical Matters. | |
| Miscellaneous. | |

